# Evaluation of the contributors of generator impedance during radiofrequency catheter ablation

**DOI:** 10.1007/s00380-025-02601-y

**Published:** 2025-10-10

**Authors:** Takayuki Sekihara, Yuma Tanaka, Yuto Ota, Koki Tanabiki, Tomohiro Yamanaka, Masaki Taniguchi, Hiroki Kawakita, Tomoaki Nakano, Akira Yoshida, Takafumi Oka, Yasushi Sakata

**Affiliations:** 1 Department of Cardiovascular Medicine, The University of Osaka Graduate School of Medicine, 2-2, Yamadaoka, Suita, Osaka 565-0871 Japan; 2 Department of Clinical Engineering, The University of Osaka Hospital, Osaka, Japan

**Keywords:** Bioelectrical Impedance, Body Mass Index, Electrodes, Hematocrit, Radiofrequency catheter ablation

## Abstract

This study aimed to clarify the contributions of dispersive electrode configuration, extracardiac impedance, and blood pool impedance to generator impedance (GI). Forty-five patients who underwent catheter ablation with Intellanav Stablepoint™ catheter were included. Four dispersive electrode positions were tested: the left hip, lower back, middle back, and upper back. For each dispersive electrode position, GI in the blood pool (BP-GI) and GI during contact with the myocardium of the left atrial anterior wall (Myo-GI) were measured at 46 kHz in standby mode. Body mass index (BMI) and hematocrit served as surrogates for extracardiac and blood pool impedance, respectively. The lowest BP-GI and Myo-GI were observed with the middle back dispersive electrode (BP-GI: 119 ± 13 Ω; Myo-GI: 123 ± 13 Ω), followed by the upper back (122 ± 13 Ω; 126 ± 13 Ω), lower back (126 ± 14 Ω; 129 ± 14 Ω), and the left hip dispersive electrode (153 ± 15 Ω; 156 ± 14 Ω). With the middle back dispersive electrode, BMI and hematocrit predicted BP-GI and Myo-GI with acceptable accuracy (adjusted *R*^2^ = 0.78 and 0.55, respectively). The standardized beta coefficients of BMI and hematocrit were 0.38 and 0.70 for BP-GI and 0.37 and 0.54 for Myo-GI, respectively. The middle back dispersive electrode yielded the lowest GI. GI differences among the back positions were small. BMI and hematocrit accurately predicted GI under the optimal (middle back) dispersive electrode position, and the effect of hematocrit was greater than that of BMI.

## Introduction

Radiofrequency (RF) current is one of the most commonly used energy sources for catheter ablation. Lesion formation during radiofrequency catheter ablation (RFCA) is influenced by various parameters, such as RF current power, contact force, the flow and sodium concentrations of irrigation water, and generator impedance (GI). GI is a composite impedance of the blood pool, myocardium, extracardiac tissue, and ablation system, including dispersive electrodes. When artificially changing the circuit impedance, a lower GI is associated with increased RF current and larger ablation lesions [1–4]. A similar result from computer simulation has also been reported [5]. In an in vivo setting, a lower GI can be achieved by modulating dispersive electrode configurations [2]. Determining the optimal dispersive electrode position with a low GI and avoiding positions with a high GI is essential to increase the efficacy of RFCA. However, the range of the GI variance caused by the dispersive electrode positioning and how far the distance between the dispersive electrode and the heart is acceptable have not been fully elucidated.

**Fig. 1:**
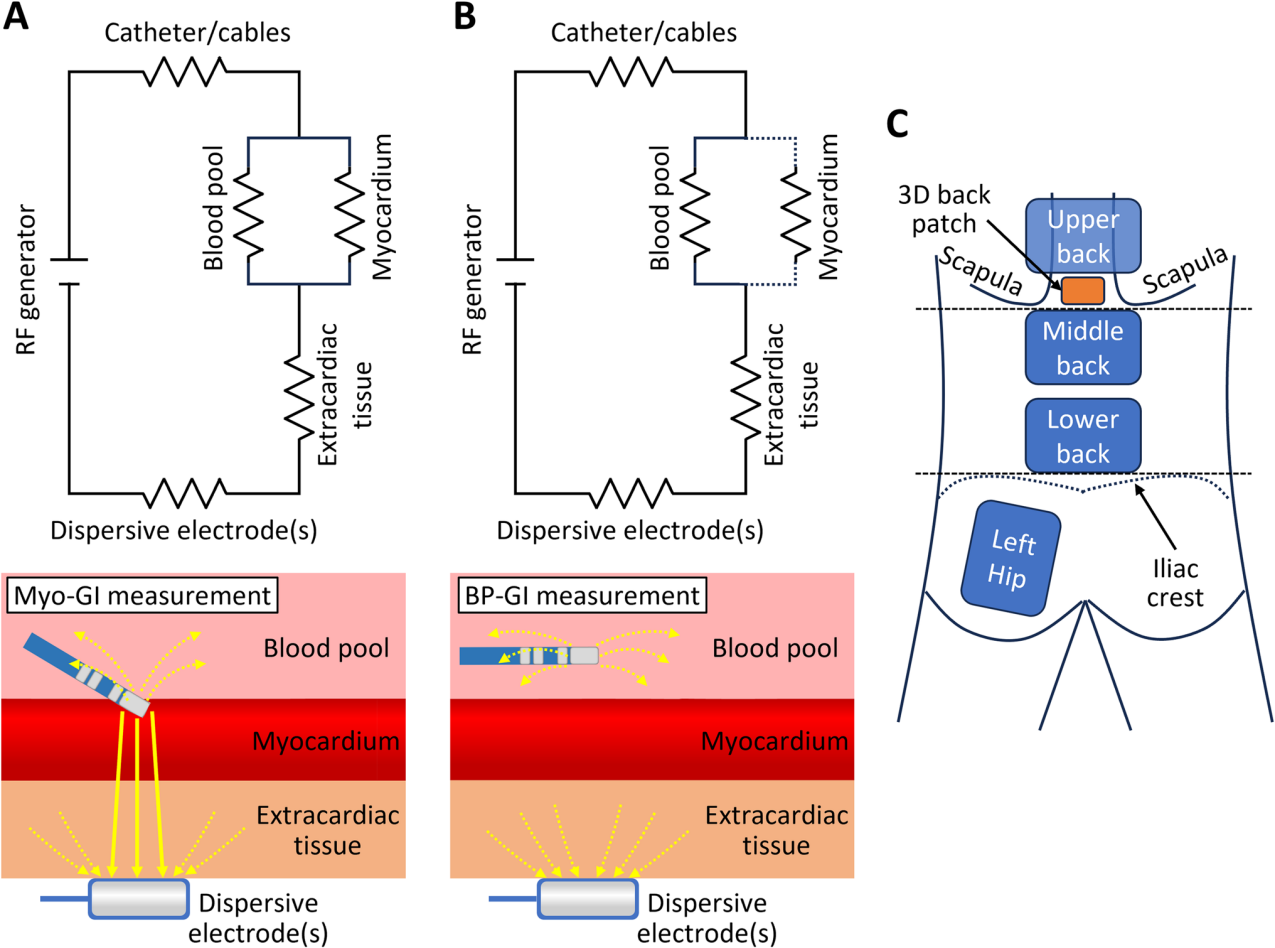
**A** and **B** Schematics of the circuit models during the measurement of Myo-GI and BP-GI based on a previous publication [6]. In the lower panels, solid and dotted arrows indicate the current across and bypassing the myocardium, respectively. **C.** Dispersive electrode positions used in this study. *BP-GI* generator impedance in the blood pool, *Myo-GI* generator impedance during contact with the myocardium

Furthermore, even though not directly modifiable, understanding the impact of a patient’s extracardiac and blood pool impedance on GI is essential to predict GI behavior. A simplified circuit model and the composite impedance of radiofrequency ablation are presented in Fig. 1 [6]. Previous studies have adopted the initial value during RF delivery as the baseline GI [1–5]. However, this baseline GI measured while the ablation catheter is in contact with the myocardium (Myo-GI) can be affected by the tightness of catheter contact or the characteristics of myocardial tissue (Fig. 1A), and thus may not be favorable for assessing the influence of dispersive electrode position, patient-specific extracardiac and blood pool impedance. On the other hand, GI in the blood pool (BP-GI) is not affected by these factors (Fig. 1B).

In this study, we aimed to clarify the contributions of dispersive electrode configuration, extracardiac impedance, and blood pool impedance to GI. First, we investigated the impact of dispersive electrode position and number on BP-GI and Myo-GI. Second, to evaluate the impact of the patient’s extracardiac and blood pool impedance on GI, we also investigated the predictability of BP-GI and Myo-GI based on the patient’s body mass index (BMI) and hematocrit (Hct) for each dispersive electrode position. BMI and Hct served as surrogates for the extracardiac and blood pool impedance, respectively, for a specific dispersive electrode position.

## Materials and methods

### Study population

This prospective, observational study included 45 patients who underwent catheter ablation requiring left atrial (LA) access using the Rhythmia HDx™ mapping system and Intellanav Stablepoint™ ablation catheter (Boston Scientific, Marlborough, MA, USA) from March 2024 to October 2024. Among them, two patients appeared in our previous publication [7]. All catheter manipulations were performed by a single operator (T.S.). This study complied with the Declaration of Helsinki, and the hospital’s institutional review board approved the study protocol. Consent was obtained by an opt-out method.

### Ablation and GI measurement

Ablation procedures were performed under deep sedation using propofol, dexmedetomidine hydrochloride, thiopental sodium, hydroxyzine hydrochloride, and pentazocine hydrochloride. Periprocedural oral anticoagulation therapy was uninterrupted. The activated clotting time was maintained at > 300 s during the procedures. Body surface electrocardiograms and bipolar intracardiac electrograms were continuously monitored and stored on a computer using a digital recording system (LABSYSTEM™ PRO; Boston Scientific). Dispersive electrodes with a conductive area of 134 cm^2^/piece (JDRP-300; Japan Lifeline, Tokyo, Japan) were connected to the ablator (Maestro 4000; Boston Scientific) throughout the study. Multiple dispersive electrodes were attached to the following positions (Fig. 1C): (1) the left hip, (2) lower back (just above the upper edge of the iliac crest), (3) middle back (just below the lower edge of the scapula), and (4) upper back (just above the lower edge of the scapula). Only the left hip, lower back, and middle back dispersive electrodes were attached to the first 11 patients. To further assess the effect of the dispersive electrode position, the upper back electrode was added to the subsequent 34 patients (from the 12th to 45th patients). Before starting ablation, the LA shell was created with IntellaMap Orion™ (Boston Scientific), and contact force zero calibration was performed. While the operator kept the ablation catheter (Intellanav Stablepoint) position stable and as far from the atrial wall as possible, BP-GI for each dispersive electrode position was measured in the above order during the standby mode (with 46 kHz of RF current [8]). Subsequently, BP-GI with dual dispersive electrodes was also measured.

For the comparison of BP-GI and Myo-GI behavior, Myo-GI measurement with a single dispersive electrode was performed in the latter 31 patients (15th to 45th patients). The LA anterior wall was selected for Myo-GI measurement because it allowed for easy maintenance of catheter contact during the measurement. Myo-GI at the LA anterior wall was measured under a stable contact force (5–10 g) to reduce the influence of catheter contact. The initial catheter position was tagged on the LA shell, and the catheter was kept at the tagged position throughout the measurement. The Myo-GI measurement was performed in the same order as BP-GI. As all four dispersive electrodes were attached before starting the session, it took only several seconds to switch the dispersive electrode position, and all BP-GI and Myo-GI measurements were performed in several minutes.

After these measurements, we subsequently performed RFCA to treat the patient’s arrhythmia. Based on the GI information acquired by the methods mentioned above, the dispersive electrode configuration was decided at the operator’s discretion.

### Statistical analysis

Continuous variables are expressed as the mean ± standard deviation or median (interquartile range). A *p-*value < 0.05 was considered statistically significant. The relationship between two continuous variables was evaluated on the basis of the correlation coefficient (*r*). Analysis of BP-GI and Myo-GI changes in each patient was performed using a paired *t*-test with Bonferroni correction: the significance level of the changes between any two dispersive electrode positions was set at 0.05/6. For the analysis of GI predictability based on BMI and Hct, multiple regression analyses were performed by the least squares method, and adjusted coefficients of determination (adjusted R^2^) and standardized beta coefficients were evaluated. All statistical analyses were performed with JMP software 14.2.0 (SAS Institute, Cary, NC, USA).

## Results

### Patients’ characteristics

Baseline patient characteristics are shown in Table 1. Most of the patients had AF with a moderately enlarged LA and normal left ventricular contraction. Average BMI and Hct were within the normal range.

**Table 1:** Baseline patient characteristics

	Overall patients (*N* = 45)	Patients with Myo-GI data (*N* = 31)	Patients without Myo-GI data (*N* = 14)
Patients’ characteristics			
Age, year	68 ± 11	68 ± 11	67 ± 13
Female	15 (33%)	11 (35%)	4 (29%)
Height, cm	164 ± 9	164 ± 9	163 ± 11
Body weight, kg	67 ± 14	68 ± 13	66 ± 14
BMI, kg/m^2^	25 ± 4	25 ± 4	24 ± 4
Hypertension	21 (47%)	14 (45%)	7 (50%)
Diabetes	5 (11%)	4 (13%)	1 (7%)
History of stroke or TIA	1 (2%)	0 (0%)	1 (7%)
CHA_2_DS_2_-VASc score	2.4 ± 1.5	2.4 ± 1.4	2.2 ± 1.6
Arrhythmia type			
Paroxysmal AF	21 (47%)	15 (48%)	6 (43%)
Non-paroxysmal AF	14 (31%)	9 (29%)	5 (36%)
Post-ablation/surgery AT	9 (20%)	6 (19%)	3 (21%)
PSVT	1 (2%)	1 (3%)	0 (0%)
Echocardiographic findings			
LVEF, %	62 ± 13	62 ± 14	63 ± 13
LA diameter, mm	42 ± 9	43 ± 8	41 ± 11
Blood test findings			
Hemoglobin (g/dL)	13.7 ± 1.6	13.6 ± 1.7	14.1 ± 1.2
Hematocrit (%)	42 ± 5	42 ± 5	42 ± 3
Attached dispersive electrodes			
Left Hip	45 (100%)	31 (100%)	14 (100%)
Lower back	45 (100%)	31 (100%)	14 (100%)
Middle back	45 (100%)	31 (100%)	14 (100%)
Upper back	34 (76%)	31 (100%)	3 (21%)

Values are presented as n (%) or mean ± standard deviation

*AF* atrial fibrillation, *AT* atrial tachycardia, *BMI* body mass index, *LA* left atrium, *Myo-GI* generator impedance during contact with the myocardium, *LVEF* left ventricular ejection fraction, *PSVT* paroxysmal supraventricular tachycardia, *TIA* transient ischemic attack

### Effect of the dispersive electrode position on BP-GI and Myo-GI

**Fig. 2:**
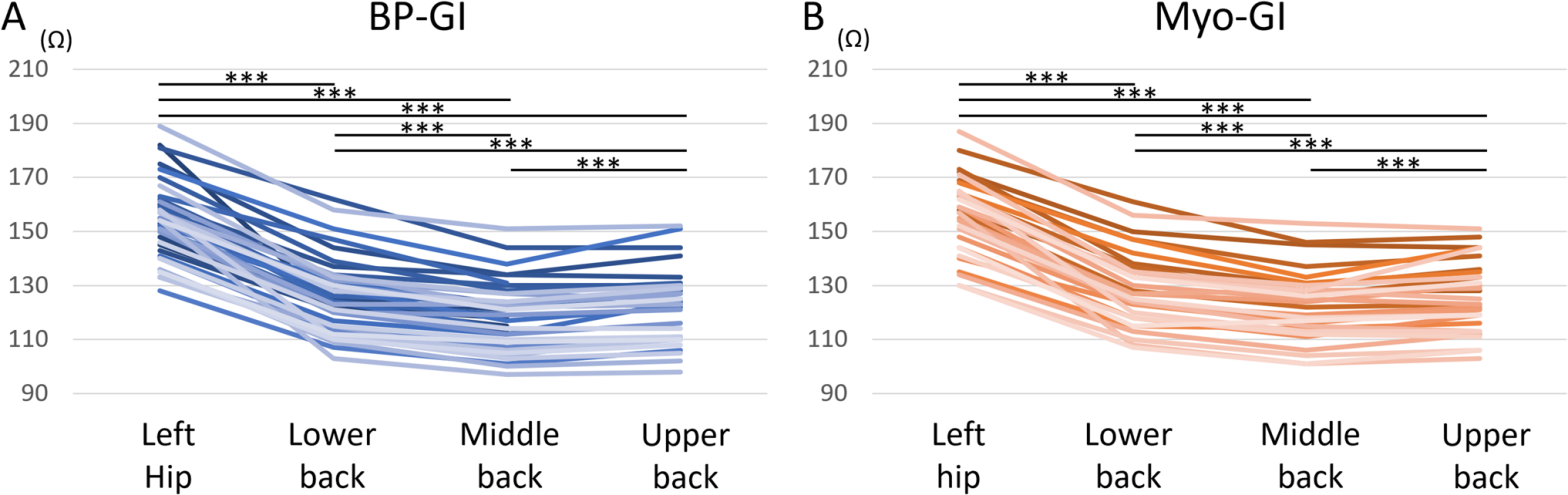
Intrapatient changes in BP-GI **A** and Myo-GI **B** when the dispersive electrode position was switched. *** *p* <.001/6. Other abbreviations are as in Fig. 1

**Fig. 3:**
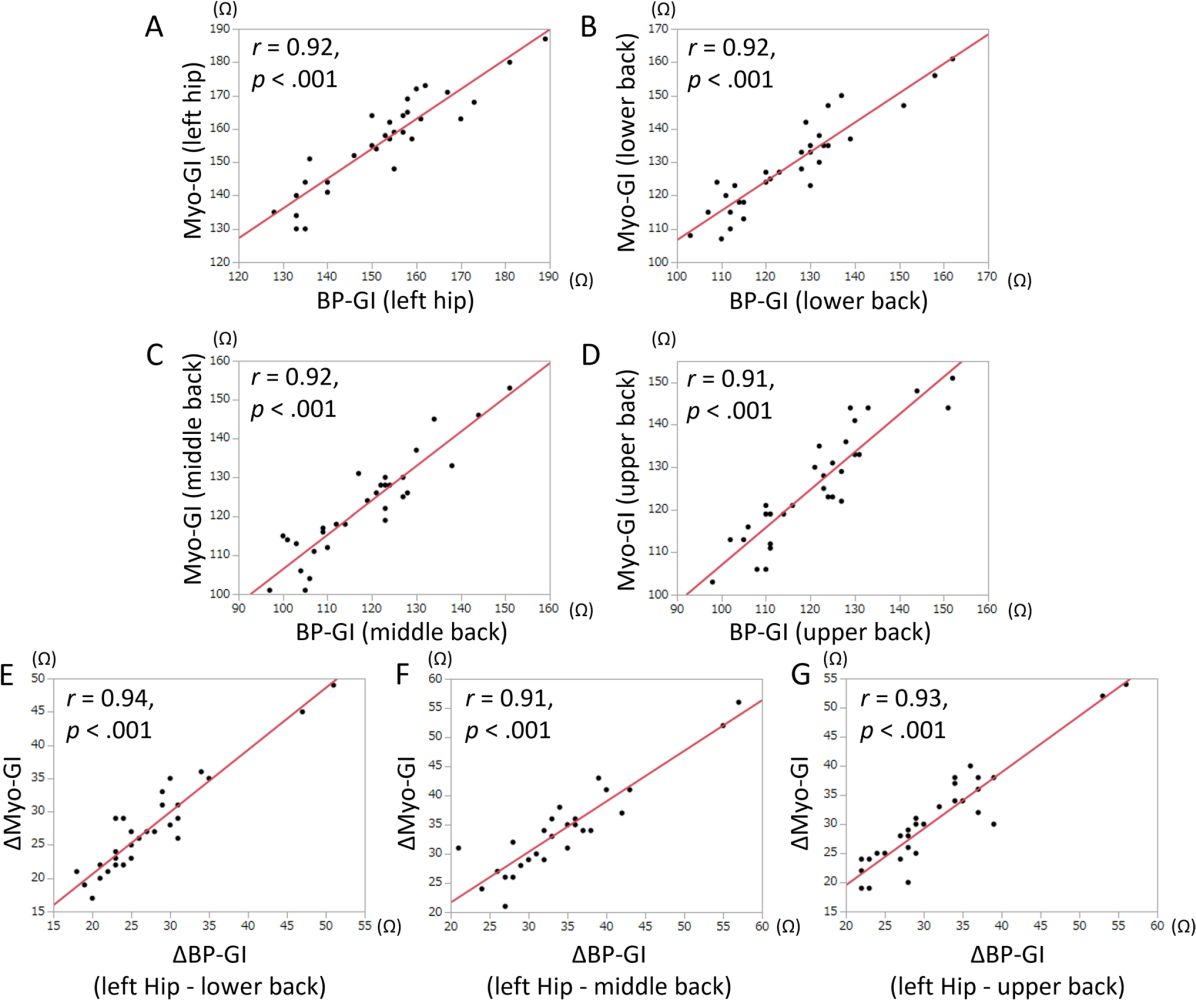
**A–D** Scatter plots of interpatient correlations of BP-GI and Myo-GI at each dispersive electrode position. **E–G** Scatter plots of intrapatient changes in BP-GI and Myo-GI while switching a dispersive electrode position. The *p-*values are based on linear regression analyses. *r* = correlation coefficient. Other abbreviations are as in Fig. 1

The mean values and intrapatient changes in BP-GI and Myo-GI when the dispersive electrode position was switched are shown in Table 2 and Figs. 2A, B. The middle back dispersive electrode position yielded the lowest BP-GI and Myo-GI, followed by the upper back position. The left hip dispersive electrode position yielded the highest BP-GI and Myo-GI. BP-GI values with dual back dispersive electrodes are shown in Table 3. Compared to a single middle back dispersive electrode, using an additional upper back dispersive electrode decreased BP-GI by 9 Ω. BP-GI and Myo-GI for each dispersive electrode position were significantly positively correlated (Figs. 3A–D). Intrapatient changes in BP-GI and Myo-GI during the dispersive electrode position switching were also significantly positively correlated (Figs. 3E–G).

**Table 2:** BP-GI and Myo-GI when switching the dispersive electrode position

	Dispersive electrode position			
	Left hip	Lower back	Middle back	Upper back
BP-GI	153 ± 15^*^	126 ± 14^*^ (−27 ± 7^*^)	119 ± 13^*^ (−34 ± 8^*^)	122 ± 13^*^ (−31 ± 8^*^)
Myo-GI	156 ± 14^†^	129 ± 14^†^ (−27 ± 7^†^)	123 ± 13^†^ (−34 ± 7^†^)	126 ± 13^†^ (−31 ± 8^†^)

The unit of measurement is ohms (Ω)

Unbracketed values are the average values of each dispersive electrode configuration, and bracketed values are the average of the changes from the left hip position

^*^Values are from the patients with the BP-GI data of all four electrode positions (34 patients)

^†^Values are from the patients with the Myo-GI data of all four electrode positions (31 patients)

*BP-GI* generator impedance in the blood pool, *Myo-GI* generator impedance during contact with the myocardium

**Table 3:** BP-GI when using dual dispersive electrodes

		Additional dispersive electrode position			
		None	Lower back	Middle back	Upper back
Baseline dispersive electrode position	Lower back	126 ± 14	N/A	113 ± 12 (− 13 ± 4)	111 ± 12 (− 15 ± 4)
	Middle back	119 ± 13	113 ± 12 (− 6 ± 2)	N/A	110 ± 12 (− 9 ± 3)
	Upper back	122 ± 13	111 ± 12 (− 11 ± 3)	110 ± 12 (− 12 ± 3)	N/A

The unit of measurement is ohms (Ω)

Unbracketed values are the average values of each dispersive electrode configuration, and bracketed values are the average of the changes from each single dispersive electrode

Values are from the patients with the BP-GI data of all back electrode positions (34 patients)

Abbreviations as in Table 2

*N/A* not applicable as these represent identical dispersive electrode positions

### Predictability of BP-GI and Myo-GI according to BMI and Hct

The results of the multiple regression analysis for BP-GI and Myo-GI are shown in Table 4. BMI and Hct predicted BP-GI and Myo-GI with acceptable accuracy (*p* < 0.001 for each model). Greater adjusted *R*^2^ values for BP-GI were achieved under dispersive electrode positions close to the LA (adjusted *R*^2^: 0.44, 0.70, 0.78, and 0.80 for the left hip, lower back, middle back, and upper back positions, respectively). The prediction formula for BP-GI with the middle back dispersive electrode position was as follows: 14.7 + 1.16 × BMI (kg/m^2^) + 1.78 × Hct (%). The same trend was observed for Myo-GI, but the adjusted R^2^ values were smaller than those for the BP-GI prediction at the corresponding dispersive electrode positions. The standardized beta for BMI was smaller than that of Hct in BP-GI and Myo-GI predictions for all dispersive electrode positions.

**Table 4:** Multiple regression analyses of BP-GI and Myo-GI based on BMI and hematocrit values

		Beta coefficient [95% CI]	Standardized beta coefficient	*p*-value
Left hip dispersive electrode	BP-GI (*N* = 45, Adjusted *R*^2^ = 0.44, *p* <.001)			
	BMI	0.76 [−0.11, 1.63]	0.21	.086
	Hematocrit	1.81 [1.08, 2.54]	0.59	.0011
	Myo-GI (*N* = 31, Adjusted R^2^ = 0.37, *p* <.001)			
	BMI	0.63 [−0.53, 1.78]	0.17	.27
	Hematocrit	1.57 [0.67, 2.47]	0.56	.0013
Lower back dispersive electrode	BP-GI (*N* = 45, Adjusted *R*^2^ = 0.70, *p* <.001)			
	BMI	0.99 [0.40, 1.58]	0.29	.0016
	Hematocrit	2.03 [1.54, 2.52]	0.72	<.001
	Myo-GI (*N* = 31, Adjusted *R*^2^ = 0.53, *p* <.001)			
	BMI	0.92 [−0.04, 1.88]	0.26	.060
	Hematocrit	1.66 [0.91, 2.41]	0.61	<.001
Middle back dispersive electrode	BP-GI (*N* = 45, Adjusted *R*^2^ = 0.78, *p* <.001)			
	BMI	1.16 [0.71, 1.60]	0.38	<.001
	Hematocrit	1.78 [1.41, 2.16]	0.70	<.001
	Myo-GI (*N* = 31, Adjusted *R*^2^ = 0.55, *p* <.001)			
	BMI	1.19 [0.33, 2.05]	0.37	.0084
	Hematocrit	1.34 [0.67, 2.01]	0.54	<.001
Upper back dispersive electrode	BP-GI (*N* = 34, Adjusted *R*^2^ = 0.80, *p* <.001)			
	BMI	1.19 [0.61, 1.76]	0.35	<.001
	Hematocrit	1.91 [1.46, 2.37]	0.72	<.001
	Myo-GI (*N* = 31, Adjusted *R*^2^ = 0.54, *p* <.001)			
	BMI	1.01 [0.12, 1.90]	0.31	.028
	Hematocrit	1.50 [0.80, 2.20]	0.59	<.001

*CI* confidence interval. Other abbreviations as in Tables 1 and 2

## Discussion

The main findings of this study are as follows: **1.** The middle back dispersive electrode position yielded the lowest BP-GI and Myo-GI, followed by the upper back and lower back positions. The GI variations among these positions were small yet statistically significant. Dual upper and middle back dispersive electrodes further lowered BP-GI by 9 Ω compared to a single middle back electrode. **2.** BMI and Hct could predict BP-GI and Myo-GI with acceptable accuracy. The predictive accuracy for BP-GI was notably higher when using the back dispersive electrode positions (adjusted *R*^2^ = 0.70–0.80) compared with the hip position (adjusted *R*^2^ = 0.44). The same trend was observed in Myo-GI predictions, although the predictive accuracy was lower than that for BP-GI. **3.** The contribution of Hct to GI was greater than that of BMI.

These findings suggest that dispersive electrode positions below or above the lower edge of the scapula (middle back and upper back positions) are optimal for attaining low GI, and the hip position should be avoided. Dual dispersive electrodes effectively decreased GI. The higher contribution of Hct compared to BMI to GI for each dispersive electrode position suggests that the impact of blood pool impedance is stronger than that of extracardiac impedance.

### Effects of dispersive electrode position on GI

Dispersive electrodes with a large surface area and proximity to the ablation sites have been reported to reduce GI [2]. In the current study, among the four dispersive electrode positions, the middle back position (just below the lower edge of the scapula) showed the lowest BP-GI and Myo-GI values, followed by the upper back position (just above the lower edge of the scapula). Dual upper and middle back dispersive electrodes further lowered BP-GI by 9 Ω compared to a single middle back electrode. The three back dispersive electrode positions showed acceptably low GI values. The 3–7 Ω difference between these positions, though statistically significant, is likely of limited clinical impact. However, despite the small differences, the reason why the middle back dispersive electrode yielded significantly smaller GI values than the other back dispersive electrodes should be considered.

The difference between the lower back and middle back positions can be simply attributed to the distance from the heart. On the other hand, the difference between the middle back and upper back positions cannot be easily explained, as both electrodes were considered to be almost equally close to the heart. Although we could not definitively elucidate the cause of the difference, we suspect that air in the lung might have increased the extracardiac impedance when the upper back dispersive electrode was used. A disproportionately high GI was observed when the hip position was used compared with the other dispersive electrode positions, possibly due to a greater amount of fat tissue in the hip than in the back.

This study also suggested the usefulness of BP-GI as a GI indicator instead of Myo-GI during dispersive electrode modulation. As described in the introduction, Myo-GI is affected by myocardial tissue characteristics and catheter contact. This point is advantageous when predicting lesion formation or the risk of steam pop at the contacting myocardium; however, it can introduce noise into the assessment of the optimal dispersive electrode position. Evaluating both types of GI may help increase the overall efficacy of RFCA.

### Predictability of GI by BMI and Hct

We also investigated the predictability of BP-GI and Myo-GI based on patient BMI and Hct because we considered that BMI and Hct could be used as indicators of extracardiac impedance and blood pool impedance in the GI measurement models (Figs. 1A and B), respectively [6]. The variability of extracardiac impedance is caused by fat-free mass, body fat, total body water, and other parameters [9]. As we could not preprocedurally collect these data, we adopted BMI as a surrogate for extracardiac impedance. Blood pool impedance is determined by Hct, electrolyte and fibrinogen concentrations, total protein, and temperature. We adopted Hct as a surrogate for blood pool impedance because Hct has been reported as a major determinant among them [10–12].

Using the middle back dispersive electrode position, identified as optimal based on the results above, the predictability of BP-GI by these two parameters was sufficiently high (adjusted *R*^2^ = 0.78). Notably, the relatively low predictability of Myo-GI (adjusted *R*^*2*^ = 0.55) with the same dispersive electrode position could be attributed to the heterogeneity of myocardial tissue characteristics and catheter contact. These results indicate that GIs with an optimal dispersive electrode configuration can be predicted before ablation.

### The contributions of dispersive electrode configuration, extracardiac impedance, and blood pool impedance on RFCA

According to the current study, dispersive electrode configuration, BMI, and Hct were demonstrated to be major determinants of GI values. The variance of GI due to these factors may affect the effectiveness of RFCA. A high GI due to an inappropriate dispersive electrode position or a high BMI value (typically related to the amount of subcutaneous fat) leads to an increase in the impedance of the overall circuit and a decrease in RF current in the myocardium. Decreased RF current in the myocardium may impair RFCA effectiveness. These points contrast with a high GI caused by tight catheter contact, which is associated with an increased risk of steam pop. Conversely, decreasing the circuit impedance by modulating the dispersive electrode configuration may contribute to offsetting the excessive extracardiac impedance and thus lead to enhancing the ablation efficacy.

A high Hct is associated with high blood impedance [10–12], which can also contribute to an increase in the impedance of the overall circuit. However, in an ex vivo experiment, Takigawa et al. reported that higher local impedance of the saline pool increased lesion depth and the rate of steam pop [13]. Theoretically, an increase in blood pool impedance (or saline pool component in an ex vivo model) can change the current distribution between the myocardium and the blood pool. Therefore, an increased GI due to high blood pool impedance may have a different effect on ablation efficacy than that caused by excessive extracardiac impedance.

### Clinical implications

According to the results of this study, the middle back dispersive electrode position, followed by the upper back position, may be optimal for lowering GI. The decrease in GI values by attaching an additional dispersive electrode was also demonstrated; additional back dispersive electrodes lowered BP-GI by 6 to 15 Ω compared to a single back dispersive electrode. This study also adds novel insight into the predictability of GI. With the dispersive electrode attached near the LA, BP-GI can be accurately predicted by BMI and Hct. A measured BP-GI greater than the predicted value possibly indicates some kind of excessive extracardiac impedance and suggests the need to modulate the dispersive electrode configuration.

## Limitations

This study has several limitations. First, we included a small number of patients, and the investigation was performed by a single operator using a single mapping and ablation system. Second, GI measurement was performed during the standby mode with 46 kHz of RF current, not during the ablation mode with 460 kHz of RF current [8]. Biological impedance is susceptible to RF frequency. Therefore, the GI behavior observed in this study might not be fully applicable to GI behavior during ablation. Third, this study did not assess the real efficacy of RFCA in relation to BP-GI and Myo-GI. We are planning to examine this issue in future investigations. Fourth, GI values with the hip dispersive electrode were somewhat higher than in the previous report [2]. As shown in Fig. 1A, GI is affected by the impedance of the ablation system. Although the qualitative results of this study may be generalized to other ablation systems, the absolute GI values and the degree of variability may depend on the ablation systems.

## Conclusions

The middle back dispersive electrode position, followed by the upper back position, may be optimal for lowering GI. Dispersive electrodes attached to the hip or below should preferably be avoided. BP-GI can be used as a GI indicator, as well as Myo-GI. Finally, BMI and Hct, the assumed surrogates for extracardiac and blood pool impedance, can predict BP-GI and Myo-GI with acceptable accuracy. These findings may contribute to assessing GI and enhancing RFCA efficacy.

## Ethical approval

The study protocol was approved by the hospital’s institutional review board. The study complied with the Declaration of Helsinki.

## Data Availability

Available upon reasonable request.

## Declarations

### Approval of the research protocol

Approved by the institutional review board (approval number: 22153).

## References

[1] Barkagan M, Rottmann M, Leshem E, Shen C, Buxton AE, Anter E (2018) Effect of baseline impedance on ablation lesion dimensions: a multimodality concept validation from physics to clinical experience. Circ Arrhythm Electrophysiol. 10.1161/CIRCEP.118.006690 30354405 10.1161/CIRCEP.118.006690

[2] Shapira-Daniels A, Barkagan M, Rottmann M, Sroubek J, Tugal D, Carlozzi MA, McConville JW, Buxton AE, Anter E (2019) Modulating the baseline impedance: an adjunctive technique for maximizing radiofrequency lesion dimensions in deep and intramural ventricular substrate: an adjunctive technique for maximizing radiofrequency lesion dimensions in deep and intramural ventricular substrate. Circ Arrhythm Electrophysiol. 10.1161/CIRCEP.119.007336 31113232 10.1161/CIRCEP.119.007336 PMC6540818

[3] Jiang X, Li S, Xiong Q, Zhang C, Peng L, Chen W, Cai Y, Yin Y, Chen S, Ling Z (2023) Effects of different ablation settings on lesion dimensions in an ex vivo swine heart model: Baseline impedance, irrigant, and electrode configuration. J Cardiovasc Electrophysiol 34:117–125 36403284 10.1111/jce.15752

[4] Bhaskaran A, Barry MA, Pouliopoulos J, Nalliah C, Qian P, Chik W, Thavapalachandran S, Davis L, McEwan A, Thomas S, Kovoor P, Thiagalingam A (2016) Circuit impedance could be a crucial factor influencing radiofrequency ablation efficacy and safety: A myocardial phantom study of the problem and its correction. J Cardiovasc Electrophysiol 27:351–357 26648095 10.1111/jce.12893

[5] Sun Y, Zhu X, Nakamura K, Wang S (2023) Evaluation of lesion characteristics and baseline impedance on high-power short-duration radiofrequency catheter ablation using computer simulation. Heart Vessels 38:1459–1467 37650926 10.1007/s00380-023-02300-6

[6] Huang SKS MJM (2019) Catheter Ablation of Cardiac Arrhythmias, 4th edn. Elsevier OHCE, Amsterdam, pp 2–8

[7] Sekihara T, Oka T, Ozu K, Yoshida A, Sakata Y (2024) Pacing cycle length-dependent electrophysiologic changes in left atrium: Poor validity of using low-voltage area and slow conduction area under specific pacing cycle length as absolute substrates of atrial fibrillation. Heart Rhythm. 10.1016/j.hrthm.2024.09.034 39304004 10.1016/j.hrthm.2024.09.034

[8] Sulkin MS, Laughner JI, Hilbert S, Kapa S, Kosiuk J, Younan P, Romero I, Shuros A, Hamann JJ, Hindricks G, Bollmann A (2018) Novel measure of local impedance predicts catheter-tissue contact and lesion formation. Circ Arrhythm Electrophysiol. 10.1161/CIRCEP.117.005831 29618475 10.1161/CIRCEP.117.005831

[9] Kyle UG, Bosaeus I, De Lorenzo AD, Deurenberg P, Elia M, Manuel Gómez J, Lilienthal Heitmann B, Kent-Smith L, Melchior JC, Pirlich M, Scharfetter H, Schols MWJ, Pichard A, ESPEN C (2004) Bioelectrical impedance analysis-part II: utilization in clinical practice. Clin Nutr 23:1430–1453 15556267 10.1016/j.clnu.2004.09.012

[10] Hirsch FG, Texter EC Jr, Wood LA, Ballard WC Jr, Horna FE, Wright IS (1950) The electrical conductivity of blood. I: Relationship to erythrocyte concentration. Blood 5:1017–1035 14791582

[11] Texter EC Jr, Hirsch FG, Horan FE, Wood LA, Ballard WC Jr, Wright IS (1950) The electrical conductivity of blood. II. Relation Red Cell Count Blood 5:1036–1048 14791583

[12] Pop GA, Chang ZY, Slager CJ, Kooij BJ, van Deel ED, Moraru L, Quak J, Meijer GC, Duncker DJ (2004) Catheter-based impedance measurements in the right atrium for continuously monitoring hematocrit and estimating blood viscosity changes; an in vivo feasibility study in swine. Biosens Bioelectron 19:1685–1693 15142603 10.1016/j.bios.2004.01.002

[13] Takigawa M, Yamamoto T, Amemiya M, Martin CA, Ikenouchi T, Yamaguchi J, Negishi M, Goto K, Shigeta T, Nishimura T, Tao S, Miyazaki S, Goya M, Sasano T (2023) Impact of baseline pool impedance on lesion metrics and steam pops in catheter ablation. J Cardiovasc Electrophysiol 34:1671–1680 37337433 10.1111/jce.15964

